# Differential Modulation of Saliva-Derived Microcosm Biofilms by Antimicrobial Peptide LL-31 and D-LL-31

**DOI:** 10.3390/pathogens12111295

**Published:** 2023-10-29

**Authors:** Kahena R. Soldati, Yaling Jiang, Bernd W. Brandt, Rob A. M. Exterkate, Mark J. Buijs, Kamran Nazmi, Wendy E. Kaman, Lei Cheng, Floris J. Bikker, Wim Crielaard, Daniela L. Zandim-Barcelos, Dong Mei Deng

**Affiliations:** 1 Department of Preventive Dentistry, Academic Center for Dentistry Amsterdam (ACTA), University of Amsterdam and Vrije Universiteit Amsterdam, 1081 LA Amsterdam, The Netherlands; soldatikahena@gmail.com (K.R.S.); y.jiang@acta.nl (Y.J.); b.brandt@acta.nl (B.W.B.); r.exterkate@acta.nl (R.A.M.E.); m.buijs@acta.nl (M.J.B.); w.crielaard@acta.nl (W.C.); 2 Department of Oral Diagnosis and Surgery, School of Dentistry at Araraquara, Universidade Estadual Paulista—UNESP, Araraquara 1680, SP, Brazil; daniela.zandim-barcelos@unesp.br; 3 State Key Laboratory of Oral Diseases & National Center for Stomatology & National Clinical Research Center for Oral Diseases & Department of Operative Dentistry and Endodontics, West China Hospital of Stomatology, Sichuan University, Chengdu 610041, China; chengleidentist@163.com; 4 Department of Oral Biochemistry, Academic Centre for Dentistry Amsterdam (ACTA), University of Amsterdam and Vrije Universiteit Amsterdam, 1081 LA Amsterdam, The Netherlands; k.nazmi@acta.nl (K.N.); w.e.kaman@acta.nl (W.E.K.); f.bikker@acta.nl (F.J.B.)

**Keywords:** saliva-derived microcosm biofilms, *Porphyromonas gingivalis*, 16S rRNA gene amplicon sequencing, oral microbiome, periodontitis

## Abstract

Microbiome modulation, aiming to restore a health-compatible microbiota, is a novel strategy to treat periodontitis. This study evaluated the modulation effects of antimicrobial peptide LL-31 and its D-enantiomer (D-LL-31) on saliva-derived microcosm biofilms, spiked with or without *Porphyromonas gingivalis*. To this end, one-day-old biofilms were incubated for 24 h with biofilm medium alone, or medium containing 40 µM LL-31 or D-LL-31, after which biofilms were grown for 5 days. Biofilms were assessed at 1 day and 5 days after intervention for the total viable cell counts, dipeptidyl peptidase IV (DPP4) activity, *P. gingivalis* amount (by qPCR) and microbial composition (by sequencing). The results showed that D-LL-31, not LL-31, significantly reduced the total viable cell counts, the *P. gingivalis* amount, and the DPP4 activity of the biofilms spiked with *P. gingivalis*, but only at 1 day after intervention. In the biofilms spiked with *P. gingivalis*, D-LL-31 tended to reduce the α-diversity and the compositional shift of the biofilms in time as compared to the control and LL-31 groups. In conclusion, D-LL-31 showed a better performance than LL-31 in biofilm modulation. The biofilm modulation function of the peptides could be impaired when the biofilms were in a severely dysbiotic state.

## 1. Introduction

Periodontitis is a biofilm-associated inflammatory disease that affects the tooth-supporting tissues and can eventually lead to tooth loss. It is highly prevalent, with 23.6% of dentate adults suffering from severe periodontitis [1]. Scaling and root planning (SRP) represents the gold-standard treatment of periodontal therapy, which aims to remove biofilms and thereby reduce inflammation [2]. When SRP fails, antibiotics or antimicrobial agents are often used as adjunctive treatments. However, the clinical efficacies of these adjunctive treatments are inconclusive [3,4,5]. Furthermore, there is a risk of acquiring antibiotic resistance when using antimicrobials for prolonged periods. Therefore, research on novel alternative adjunctive therapies is urgently needed.

Microbiome modulation therapy has been proposed as a potential adjunctive treatment to SRP [6]. The concept of this therapy is derived from the notion that periodontitis results from a shift of subgingival microbiota from symbiosis to dysbiosis, in which a group of periodontitis-associated bacterial species become enriched and trigger inflammation [6]. It is believed that the keystone pathogens, such as *Porphyromonas gingivalis*, orchestrate this unfavorable shift along with accessory pathogens, such as *Parvimonas micra*, whose pathogenic potential only becomes evident in the context of a heterotypic microbial community [7,8,9]. The goal of microbiome modulation therapy is to re-establish a healthy host–microbe balance by targeting the drivers of community dysbiosis.

Antimicrobial peptides (AMPs) are a group of diverse and bioactive small-molecular-weight molecules with broad-spectrum antimicrobial activity. Most AMPs can be found in eukaryotic and prokaryotic cells as a part of a host defense system [10]. Their primary mode of action against bacteria is by direct disruption and lysis of bacterial membranes via interaction between the negatively charged phospholipids of cell membrane and the positively charged AMPs. As compared to broad-spectrum antibiotics, AMPs are weaker in their antimicrobial activities but better in microbiome modulation and in mediating the interaction between microbiome and innate immune system [11]. It was shown that mice deficient in ⍺-defensin, a type of AMP, had a decreased abundance of Bacteroidota and an increased abundance of Bacillota as compared to those of wild-type mice, even though the total bacterial numbers were similar in both types of mice [12]. Furthermore, AMPs secreted by epithelium have been shown to shape the gut microbiota and determine healthy or pathological phenotypes [13].

Cathelicidin LL-37 is a well-known human AMP. It is a cationic peptide consisting of 37 amino acids with two leucyl residues at its N terminus, and it is widely distributed in human saliva, skin, gastrointestinal tract, urinary tract and respiratory airways [14]. A recent study showed that a defective cathelicidin-related antimicrobial peptide (CRAMP) expression in the colon of newborn nonobese diabetic mice could result in gut microbial dysbiosis and promote auto-immune response. Local treatment with LL-37 restored colonic homeostasis and prevented auto-immune diabetes [15]. In periodontitis patients, LL-37 deficiency has been associated with increased susceptibility to periodontitis [16]. It was hypothesized that a lack of LL-37 might instigate an impaired control over the growth of periodontal pathogens in the subgingival biofilms, thus leading to the breakdown of microbial homeostasis and a compositional shift towards dysbiosis [17,18]. Therefore, LL-37 might be a potent candidate for modulating the oral microbiome. Since LL-37 is a relatively long peptide and the synthesis cost might be challenging for a wide application, efforts have been made to truncate the peptide without affecting its function [14]. LL-31 is a truncated variant of LL-37 lacking the 6 C-terminus amino acid residues. It exhibited the strongest antimicrobial activity among tested LL-37 fragments [19]. Since the presence of D-amino acids greatly improves the proteolytic stability of peptides [20], a D-enantiomeric form of LL-31, D-LL-31, was synthesized which showed a stable and significant antimicrobial performance [21].

Although the examples mentioned above presented a few successful microbiome modulations, our knowledge of this therapy is still in infancy. It is not clear which factor(s) could influence the efficacy of the therapy. The aims of this study are twofold: (1) to compare the microbiome modulation effect between peptide LL-31 and D-LL-31; (2) to investigate whether the growth and the dysbiotic state of microbiota could influence the peptide function. To this end, we cultured saliva-derived microcosm biofilms using a serum-rich medium, spiked with and without *P. gingivalis*, creating different dysbiotic states. A previous study has shown that the *P. gingivalis*-enriched biofilm model resembled the in vivo pathogen-enriched subgingival microbiota in patients with severe periodontitis [22].

## 2. Materials and Methods

### 2.1. Saliva Collection

Saliva was collected from 10 donors who were systemically healthy and had no active oral diseases such as caries and periodontal disease. They had not taken antibiotics within at least 3 months before saliva donation. The Medical Ethical Committee of the VU University Medical Center Amsterdam approved the study protocol (document number 2011/236). Donors were asked not to perform any oral hygiene for 24 h and to refrain from food or drink intake for at least 2 h prior to saliva donation. Unstimulated saliva from 10 donors was pooled together, mixed in sterile glycerol (final concentration 30%), and stored at −80 °C until use.

### 2.2. Peptide Synthesis

The peptide LL-31 (LLGDFFRKSKEKIGKEFKRIVQRIKDFLRNL) and its D-form (D-LL-31) were synthesized and purified, as has been described previously [23]. Briefly, the peptides were synthesized by a solid-phase peptide synthesis using fluoren-9-ylmethoxycarbonyl (Fmoc) chemistry with a Siro II synthesizer (Biotage, Uppsala, Sweden) and were purified by HPLC on a Dionex Ultimate 3000 system (Thermo Scientific, Breda, The Netherlands). Mass spectrometry with a Microflex LRF MALDI-TOF (Bruker Daltonik GmbH, Bremen, Germany) was used to confirm the authenticity of the peptides [24]. The peptides were reconstituted in Milli-Q water (MQ) to 10 mM and stored at −20 °C until use.

### 2.3. Bacterial Strains and Growth Conditions

The bacterial strains used in this study were *P. gingivalis* ATCC 33277, *Fusobacterium nucleatum* ATCC 10953 and *Streptococcus mitis* LMG 14557. All 3 strains were tested in a growth inhibition assay. *P. gingivalis* was further tested in biofilm experiments.

*P. gingivalis* and *F. nucleatum* were routinely maintained on trypticase soy agar plates containing 5% sheep’s blood, 5 µg/mL hemin and 1 µg/mL menadione (BA plates) under anaerobic condition (10% CO_2_, 10% H_2_ and 80% N_2_) at 37 °C. For planktonic cultures, *P. gingivalis* and *F. nucleatum* were grown in brain–heart infusion broth supplemented with 10 µg/mL hemin and 1 µg/mL menadione (BHI-HM) anaerobically at 37 °C. *S. mitis* was grown in BHI broth supplemented with 1% glucose (BHI-G) anaerobically at 37 °C.

A complex Thompson medium (TP) supplemented with 10% heat-inactivated fetal bovine serum was used for biofilm growth [25]. This medium was chosen because it has been shown to support the growth of a diverse oral bacterial community and could mimic the nutritional environment of periodontal pocket [25].

### 2.4. Bacterial Growth Inhibition by LL-31 and D-LL-31

The growth inhibitory concentrations of LL-31 and D-LL-31 were first determined using planktonic bacterial cultures. In brief, the full-grown cultures of *P. gingivalis*, *F. nucleatum* and *S. mitis* were diluted to OD600 of 0.02 (approximately 2 × 10^7^ CFU/mL bacterial cells) in fresh medium (BHI-HM or BHI-G). LL-31 and D-LL-31 were then added to each bacterial dilution to reach the final concentrations of 20, 40 and 80 µM. The mixtures were grown anaerobically at 37 °C for 24 h, after which OD600 values were recorded using a spectrometer (SpectraMax M2; Molecular Devices, Sunnyvale, CA, USA). MQ was added to the bacterial dilutions as the negative control.

### 2.5. Microcosm Biofilm Formation and Peptide Intervention

**Figure 1:**
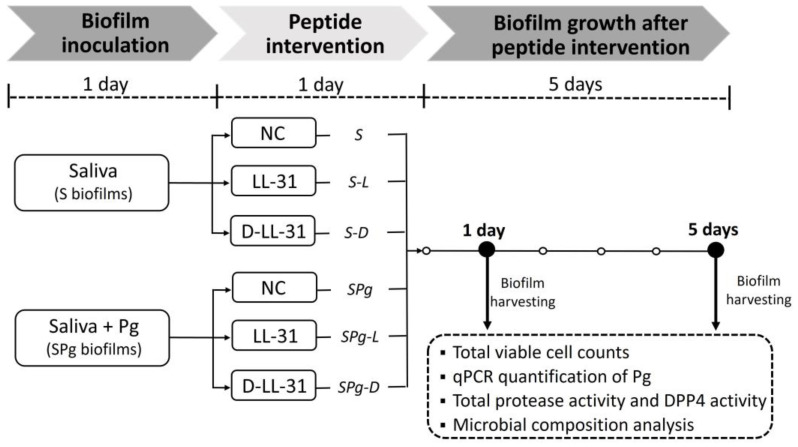
Experimental scheme. Pg, *P. gingivalis*; S biofilms, saliva-derived microcosms alone; SPg biofilms, saliva-derived microcosms spiked with *P. gingivalis*. Biofilms were treated with peptide LL-31 (40 µM), peptide D-LL-31 (40 µM), or MQ water (negative control, NC).

The microcosm biofilms, spiked with or without *P. gingivalis*, were cultured in an Amsterdam Active Attachment model (AAA-model) assembled with 9.5-mm hydroxyapatite (HA) discs (HIMED, Old Bethpage, NY, USA) for 1 day and then treated with LL-31 or D-LL-31 for 1 day. These treated biofilms were further grown for 5 days without any intervention. Figure 1 depicts the scheme of biofilm growth and peptide intervention.

For biofilm inoculation, the full-grown *P. gingivalis* pre-culture was centrifuged and then the cell pellets were resuspended in fresh TP medium. Subsequently, the glycerol stock of the pooled saliva was diluted to a cell density of 4 × 10^6^ CFUs/mL in either fresh TP medium (S biofilms) or the abovementioned *P. gingivalis* resuspensions which contained 1 × 10^9^ CFUs/mL of *P. gingivalis* (SPg biofilms). Both mixtures were inoculated (1.3 mL/well) into the 24-well AAA-model and incubated anaerobically at 37 °C for 1 day to achieve initial microbial attachments.

The HA discs with the 1-day old microcosm biofilms were transferred to the TP media containing 40 µM LL-31, 40 µM D-LL-31, or MQ (control) and incubated anaerobically at 37 °C for 1 day. Mucin and serum were left out from the TP medium to avoid peptide inactivation or precipitation.

After the 1-day peptide intervention, HA discs with biofilms were rinsed with MQ and were then transferred to fresh TP for further incubation. The biofilm medium was refreshed daily. One day and 5 days after peptide intervention, biofilms were harvested and subjected to different analysis. Biofilm cell pellets were used for total viable cell counts, dipeptidyl peptidase IV (DPP4) activity determination and genomic DNA (gDNA) isolation for quantitative PCR (qPCR) and 16S rRNA gene amplicon sequencing. In addition, biofilm spent medium was used for measuring the total protease activity of biofilms.

The experiment was repeated 3 times, and in each experiment, triplicate biofilm samples for each group at each time point were included.

### 2.6. Total Viable Cell Counts

Each HA disc with biofilm was removed from the AAA-model and transferred into 2 mL of cysteine peptone water (CPW, pH 7.2). The biofilms were dispersed by vortexing for 30 s, after which the samples were sonicated on ice at a 1 s pulse at an amplitude of 40 W (Vibra Cell; Sonics & Materials Inc., Newtown, CT, USA) for 2 min. Fifty microliters of the dispersed biofilm samples were serially diluted and plated on BA plates. The BA plates were incubated for 7 days at 37 °C under anaerobic conditions, after which total viable cell counts were determined by counting the colony-forming units (CFUs) on each plate.

The remaining dispersed biofilm samples were centrifuged, and the biofilm cell pellets were stored at −80 °C for DPP4 activity determination and gDNA isolation.

### 2.7. Total Protease Activity and DPP4 Activity

The influences of peptide LL-31 and D-LL-31 on the protease activity of biofilms were assessed by the total protease and DPP4 activities at both 1 day and 5 days after peptide intervention.

Total protease activity of biofilms was measured based on the cleavage of a broad-spectrum fluorescence resonance energy transfer substrate PEK-054 [26]. In brief, biofilm spent medium was filtered (0.2 μm pore size) to remove biofilm cells and diluted at a ratio of 1:10 with PBS (pH 7.4). The dilutions were mixed with 16 μM PEK-054 and added into a black, clear-bottom 96-well plate (Greiner Bio-One, Frickenhausen, Germany) (50 µL/well). Fresh TP was included as the medium control. Fluorescence was recorded for 1 h at 37 °C with 2-min intervals in a fluorimeter (Fluostar Galaxy, BMG Laboratories, Offenburg, Germany) with an excitation wavelength of 485 nm and an emission wavelength of 530 nm. Relative fluorescence (RF) values, as RF per minute (RF/min), were obtained after correction against values of the medium control.

DPP4 activity of the biofilms was determined using a fluorogenic substrate, glycylprolyl-7-amino-4-methylcoumarin (Gly-Pro-AMC) (AAT Bioquest Inc., Pleasanton, CA, USA). Briefly, biofilm cell pellets were re-suspended with 10 mM Tris buffer (pH 7.6) and co-incubated with 50 μM Gly-Pro-AMC substrate at 37 °C for 1 h, after which the fluorescence of each sample was recorded at 380-nm excitation and 500-nm emission wavelength. DPP4 activity findings of samples were presented as RF values which were corrected against values of the background control group, where the substrate was added to 10 mM Tris buffer only.

### 2.8. gDNA Isolation and Quantification of P. gingivalis

The gDNA in the biofilm samples was isolated according to the established protocols in our lab. In brief, biofilm cell pellets were resuspended in Tris-EDTA buffer and were added into wells of a 96-deep-well plate containing Tris-saturated phenol, 0.1 mm zirconium beads and Mag lysis buffer (LGC Genomics, Berlin, Germany). Samples were mechanically lysed by bead-beating for 2 min at 2100 oscillations/min in a Mini-BeadBeater-96 (BioSpec Products, Bartlesville, OK, USA). Subsequently, gDNA was purified using the Agowa Mag mini DNA extraction kit (LGC Genomics, Berlin, Germany).

To determine the amount of *P. gingivalis* in biofilms, a species-specific qPCR was performed. The reaction mixture contained primers/probe targeting the 16S rRNA gene of *P. gingivalis* [27], LightCycler 480 Probes Master mix (Roche Diagnostics, Basel, Switzerland) and gDNA template in a total reaction volume of 20 µL. qPCR was performed with the LightCycler 480-II (Roche Diagnostics) using the following procedure: 5 min pre-incubation at 95 °C, followed by 45 amplification cycles at 95 °C for 10 s (denaturation) and 60 °C for 20 s (annealing and extension). The concentration (ng/μL) of *P. gingivalis* DNA in samples was calculated from a standard curve generated using pure gDNA of *P. gingivalis* ATCC 33277.

### 2.9. 16S rRNA Gene Amplicon Sequencing and Data Processing

The 16S rRNA gene amplicon sequencing was conducted to determine the microbial composition of the biofilms following an in-house protocol. Briefly, the V4 hypervariable region of the 16S rRNA gene was amplified with barcoded forward and reverse primers. The generated amplicons were pooled equimolarly and purified from agarose gel. From the amplicon mix, 8 pmol including 30% PhiX was loaded into the flow cell and sequencing of the amplicons (2 × 251-bp length) was conducted on the Illumina MiSeq platform at the Tumor Genome Analysis Core of Amsterdam UMC (Amsterdam, The Netherlands), using the MiSeq reagent kit V3 (Illumina, Inc., San Diego, CA, USA). The paired-end reads were merged, quality-filtered and clustered into operational taxonomic units (OTUs) at 97% similarity. The representative (most abundant) sequence of each OTU was assigned a taxonomy using the ribosomal database project (RDP) classifier [28] and the *Human Oral Microbiome Database* (HOMD) version 14.51 [29].

### 2.10. Data Analysis and Statistics

To examine the effects of peptides on the viable cell counts, amount of *P. gingivalis*, total protease activity and DPP4 activity of the biofilms per collection time point, one-way ANOVA followed by a Bonferroni post hoc test was performed in SPSS version 25 (SPSS Inc., Chicago, IL, USA). CFU data were log_10_ transformed prior to analysis. Differences were considered statistically significant if *p* < 0.05.

For sequencing data analysis, the OTU table was randomly subsampled at 6000 reads per sample. The species richness (the number of OTUs per sample) and the Shannon diversity index were calculated using PAST (Paleontological Statistics) software version 4.05 [30]. The effects of peptides on these two variables were analyzed with one-way ANOVA followed by a Bonferroni post hoc test. The OTU table was log_2_ transformed for ordination using principal component analysis (PCA) in PAST. Statistical differences in the microbial profiles of the biofilms in different groups at each time point were assessed using one-way permutational multivariate analysis of variance (PERMANOVA) with the Bray–Curtis similarity index and 9999 permutations. To identify differentially abundant OTUs, the linear discriminant analysis (LDA) effect size (LEfSe) biomarker discovery tool was used [31]. Only OTUs with a relative abundance higher than 0.01% on the entire subsampled dataset were included in LEfSe analysis. To identify OTUs that were differentially abundant by biofilm age, the dataset was first split by biofilm type (S or SPg biofilms) and then analyzed separately, using biofilm age as class and peptide treatment as subclass. Default settings were used, except that the LDA threshold was set to 4 and pairwise comparisons were performed only among the subclasses with the same name. In addition, differentially abundant OTUs caused by peptide treatments was analyzed on the S or SPg biofilms from 5 days after peptide treatment. Default settings were used except that the LDA threshold was set to 4 and one-against-all was used as the strategy for multi-class analysis in this case.

## 3. Results

### 3.1. Bacterial Growth Inhibition by LL-31 and D-LL-31

**Figure 2:**
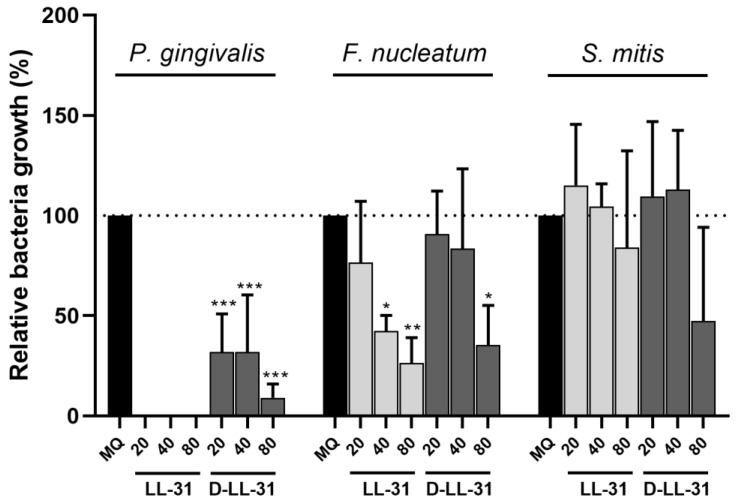
The influences of LL-31 and D-LL-31 on the growth of *P. gingivalis*, *F. nucleatum* and *S. mitis* in planktonic cultures. Peptides were tested at concentrations of 20, 40 and 80 µM. Data are presented as mean ± standard deviation of percentage bacterial growth (%), relative to the negative control group (MQ water). Statistically significant differences as compared to the negative control group are indicated by asterisks: * *p* < 0.05; ** *p* < 0.005; *** *p* < 0.0005 (one-way ANOVA followed by Bonferroni post hoc test).

The influences of LL-31 and D-LL-31 on the growth of the planktonic cultures of *S. mitis*, *F. nucleatum* and *P. gingivalis* are shown in Figure 2. LL-31 led to a complete growth inhibition of *P. gingivalis*, even at 20 µM. Although D-LL-31 was less potent than LL-31, it resulted in a more than 50% growth inhibition at the concentrations of 20 and 40 µM. Such differential growth inhibition of LL-31 and D-LL-31 could also be observed for *F. nucleatum*: while LL-31 significantly inhibited its growth from 40 µM, a D-LL-31 concentration of 80 µM was needed to achieve similar growth inhibition. As for *S. mitis*, no significant growth inhibition was observed at the tested concentrations of both peptides.

### 3.2. Total Viable Cell Counts and the Amounts of P. gingivalis in Biofilms

**Figure 3:**
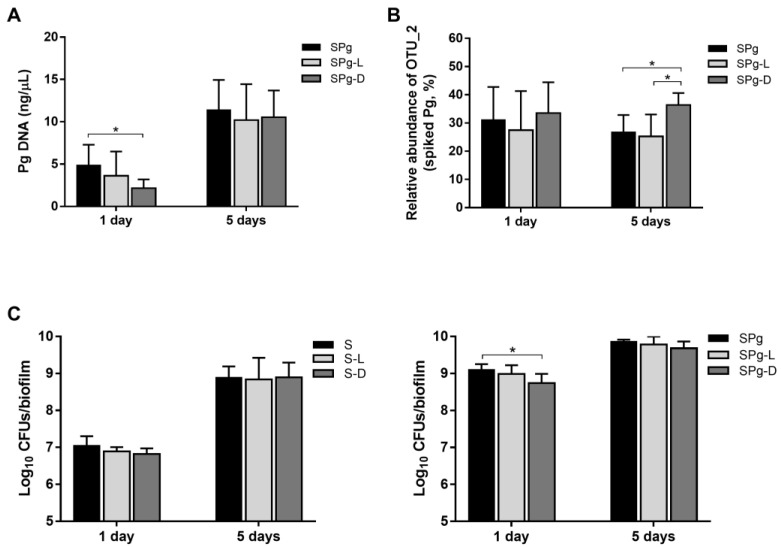
(**A**) The amount of *P. gingivalis* in the SPg biofilms at 1 day and 5 days after peptide intervention as determined by qPCR; data are presented as the calculated DNA concentration (ng/µL) of *P. gingivalis*. (**B**) Relative abundance of OTU_2 in SPg biofilms at 1 day and 5 days after peptide interventions. The representative sequence of OTU_2 was confirmed to be identical to the spiked strain *P. gingivalis* ATCC 33277 by blasting against the expanded HOMD database (http://www.homd.org (accessed on 23 December 2022); HOMD 16S rRNA RefSeq version 15.22) using default parameters. (**C**) Total viable-cell counts of S biofilms (left) and SPg biofilms (right) at 1 day and 5 days after peptide interventions. Data are represented as log_10_ CFU counts. S biofilms, saliva-derived microcosms alone; SPg biofilms, saliva-derived microcosms spiked with *P. gingivalis*; L, intervention by peptide LL-31; D, intervention by peptide D-LL-31. All data represent mean ± standard deviation of three independent experiments. Statistically significant differences between different groups at each time point are indicated by asterisks: * *p* < 0.05 (one-way ANOVA followed by Bonferroni post hoc test).

Next, we investigated whether LL-31 and D-LL-31 at 40 µM were able to modulate a microbial community, and in particular the *P. gingivalis*-enriched microbiota. Figure 3A shows the amount of *P. gingivalis* in the *P. gingivalis*-spiked microcosm biofilms (SPg biofilms, including SPg, SPg-L and SPg-D groups), which was quantified by a species-specific qPCR probe. As compared to the control group (SPg: 4.87 ± 2.43 ng/µL), only D-LL-31 was able to significantly lower the amount of *P. gingivalis* (SPg-D: 2.16 ± 0.25 ng/µL) at 1 day after intervention. However, this inhibitory effect diminished at 5 days after intervention and the amount of *P. gingivalis* was similar among the three groups. In addition, a twofold increase in the amount of *P. gingivalis* could be seen in all three groups from 1 day to 5 days. No *P. gingivalis* was detected in the saliva-derived microcosms alone (S biofilms, including S, S-L and S-D groups). Hence data of these groups were not shown.

Figure 3B shows the relative abundance of OTU_2, calculated based on sequences, whose representative sequence was confirmed to be identical to that of the spiked strain *P. gingivalis* ATCC 33277, in the SPg biofilms. The relative abundance of the spiked *P. gingivalis* reached 30% in the SPg biofilms at 1 day after intervention and remained at this level. In contrast to qPCR results, D-LL-31 resulted in a higher relative abundance of *P. gingivalis* at 5 days after intervention than was determined for the other two groups. There was no sequence of the spiked *P. gingivalis* strain in the S biofilms.

In terms of total viable cell counts, again, D-LL-31, not LL-31, significantly reduced the viable counts of SPg biofilms (Figure 3C, right) at 1 day after intervention as compared to the control group. But this reduction was diminished 5 days after intervention. In the S biofilms (Figure 3C, left), peptide treatments did not affect the biofilm viability. Furthermore, the total cell counts in both the S and SPg biofilms increased significantly with time, irrespective of the peptide used. The total cell counts of SPg biofilms were 1–2 log higher than those of S biofilms at each corresponding biofilm collection point (1 day after intervention: 9.10 ± 0.15 vs. 7.05 ± 0.25 log_10_ CFUs/biofilm; 5 days after intervention: 9.86 ± 0.05 vs. 8.89 ± 0.30 log_10_ CFUs/biofilm).

### 3.3. Total Protease Activity and DPP4 Activity of the Biofilms

**Figure 4:**
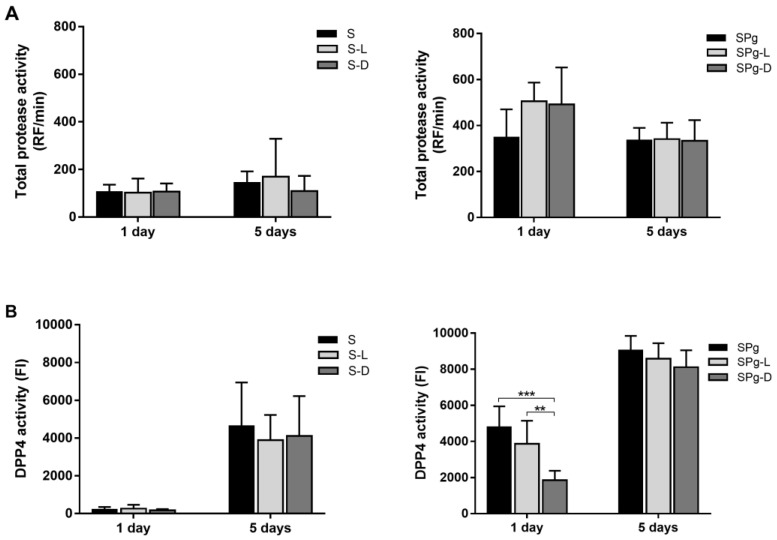
(**A**) Total protease activity of S biofilms (left) and SPg biofilms (right) at 1 day and 5 days after peptide interventions; data are presented as relative fluorescence (RF) values per min; (**B**) DPP4 activity of S biofilms (left) and SPg biofilms (right) at 1 day and 5 days after peptide interventions; data are presented as fluorescence intensity (FI) values. S biofilms, saliva-derived microcosms alone; SPg biofilms, saliva-derived microcosms spiked with *P. gingivalis*; L, intervention by peptide LL-31; D, intervention by peptide D-LL-31. All data represent mean ± standard deviation of three independent experiments. Statistically significant differences between different groups at each time point are indicated by asterisks: ** *p* < 0.005; *** *p* < 0.0005 (one-way ANOVA followed by Bonferroni post hoc test).

Total protease activity of the biofilms was measured using a fluorescence resonance energy transfer probe PEK-054. In general, the total protease activity of SPg biofilms (Figure 4A, right) was considerably higher than that of S biofilms (Figure 4A, left). However, the activity was similar among the three groups within each type of biofilms, indicating that the peptide treatments did not affect the total protease activity of the biofilms.

DPP4 is a clinical periodontal biomarker that has been shown to be positively correlated with the prevalence of *P. gingivalis* as well as disease severity in periodontitis patients [32,33]. Figure 4B displays the DPP4 activity of the biofilms. Again, D-LL-31, not LL-31, significantly reduced the DPP4 activity of SPg biofilms (Figure 4B, right) at 1 day after peptide treatments as compared to the control group. This reduction was diminished at 5 days after peptide treatments. Moreover, DPP4 activities of all SPg biofilms significantly increased over time. Interestingly, the DPP4 activities of S biofilms (Figure 4B, left) were hardly detectable at 1 day after intervention, but were significantly increased at 5 days after intervention, even though they were always lower than those of the corresponding SPg groups.

### 3.4. Major Bacterial Genera and α-Diversity of the Biofilms

16S rRNA gene amplicon sequencing was conducted to examine the effect of peptide interventions on biofilm composition. A total number of 221 distinct OTUs were identified from the sequence data after subsampling at equal depth of 6000 reads per sample. After subsampling, a total of 12 samples of S biofilms were excluded from downstream analysis because the reads of these samples were lower than 6000. Consequently, the 1-day S, S-L and S-D groups contain seven, three and five samples, respectively.

**Figure 5:**
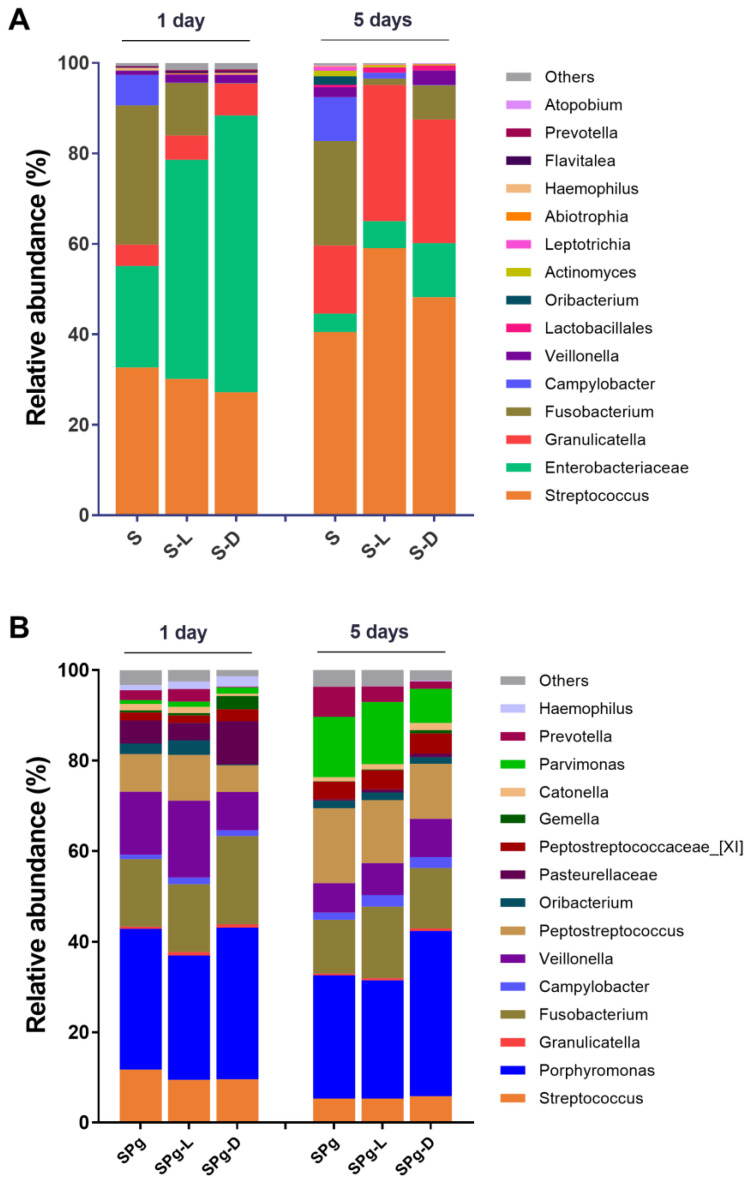
Relative abundance of top 15 most abundant bacterial genera or higher taxa (remaining genera are grouped as “Others”) in: (**A**) S biofilms; (**B**) SPg biofilms, at 1 day and 5 days after peptide intervention. Data represent an average of replicate samples of three independent experiments. S biofilms, saliva-derived microcosms alone; SPg biofilms, saliva-derived microcosms spiked with *P. gingivalis*; L, intervention by peptide LL-31; D, intervention by peptide D-LL-31.

Figure 5 shows an overview of the relative abundance of the top 15 most abundant bacterial genera or higher taxa in biofilms. The two types of biofilms, spiked with or without *P. gingivalis*, had distinct microbial composition irrespective of peptide interventions. The S biofilms were dominated by *Streptococcus*, *Enterobacteriaceae* and *Granulicatella* (Figure 5A), whereas the SPg biofilms were dominated by *Porphyromonas* and *Fusobacterium* (Figure 5B). Biofilm age had a greater effect on biofilm microbial composition than did the peptide treatments. For example, the relative abundance of *Enterobacteriaceae* decreased while that of *Granulicatella* increased as the S biofilms aged (Figure 5A); also, the relative abundance of *Pasteurellaceae* decreased while that of *Parvimonas* increased as the SPg biofilms aged (Figure 5B).

**Figure 6:**
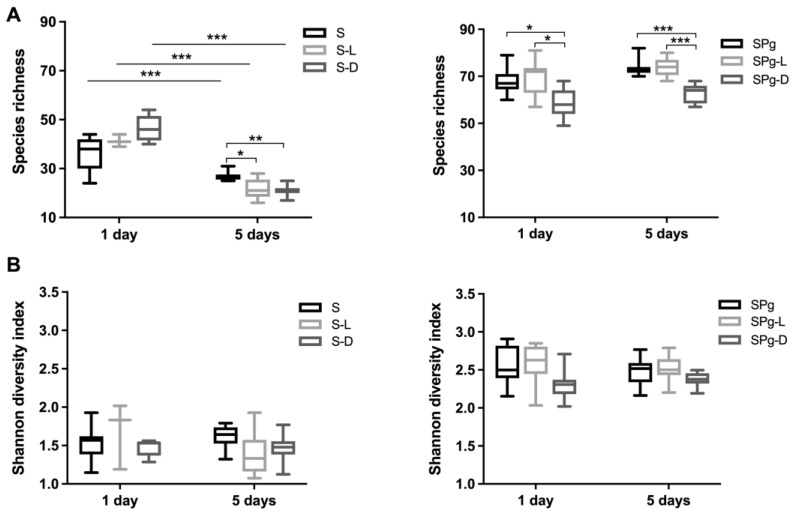
The α-diversity analyses of biofilms: (**A**) Species richness of S biofilms (left) and SPg biofilms (right) at 1 day and 5 days after peptide interventions; (**B**) Shannon diversity index of S biofilms (left) and SPg biofilms (right) at 1 day and 5 days after peptide interventions. S biofilms, saliva-derived microcosms alone; SPg biofilms, saliva-derived microcosms spiked with *P. gingivalis*; L, intervention by peptide LL-31; D, intervention by peptide D-LL-31. Statistically significant differences are indicated by asterisks: * *p* < 0.05; ** *p* < 0.005; *** *p* < 0.0005; one-way ANOVA followed by Bonferroni post hoc test was used for comparison between different groups within each time point; two-way ANOVA followed by Bonferroni post hoc test was used for comparison between time points.

The α-diversity (Figure 6), indicated by both species richness and the Shannon index, was twofold higher in SPg biofilms than in S biofilms. However, they did not change as biofilm aged, except that the species richness of S biofilms significantly decreased over time. The peptide treatments only affected the species richness, not the Shannon index (Figure 6A). Compared to the control groups, D-LL-31 significantly reduced the species richness of S biofilms (5 days) and SPg biofilms (1 day and 5 days). LL-31 also significantly reduced the species richness of S biofilms (5 days).

### 3.5. Microbial Composition of the Biofilms

**Figure 7:**
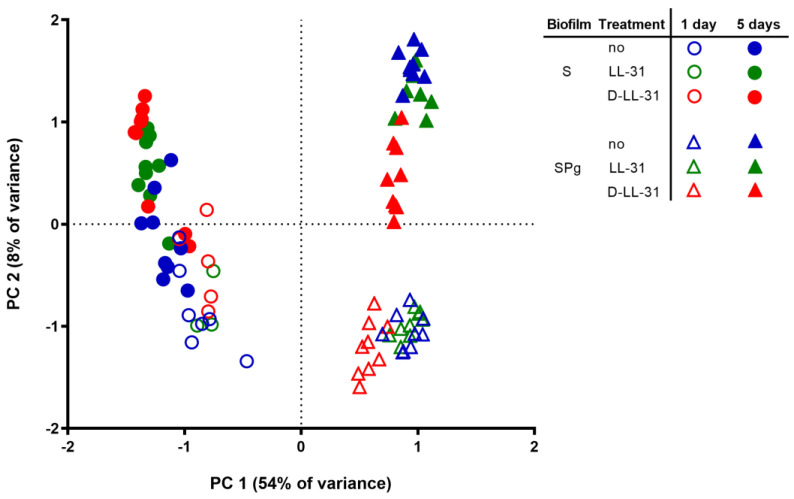
Principal component analysis (PCA) plot of S biofilms (symbols in circles) and SPg biofilms (symbols in triangles) at 1 day and 5 days after peptide interventions. S biofilms, saliva-derived microcosms alone; SPg biofilms, saliva-derived microcosms spiked with *P. gingivalis*; L, intervention by peptide LL-31; D, intervention by peptide D-LL-31.

The principal component analysis (PCA) plot (Figure 7) showed that the microbial compositions of both types of biofilms were considerably altered by the spiking of *P. gingivalis* (PC 1) and the biofilm’s age (PC 2). The LL-31 and D-LL-31 peptide treatments also altered the biofilm composition, but to a lesser extent. The significance levels of the LL-31 and D-LL-31 effects are demonstrated by means of one-way PERMANOVA analysis (Table 1). In S biofilms, LL-31 and D-LL-31 significantly altered biofilm composition as compared to the control group 5 days after the treatments (LL-31 treatment: *F* = 3.5, *p* = 0.008; D-LL-31 treatment: *F* = 5.4, *p* = 0.002); in SPg biofilms, only D-LL-31 significantly altered the biofilm composition as compared to the control and LL-31 treatment groups from 1 day after treatment (*F* > 5.6, *p* < 0.01).

**Figure 8:**
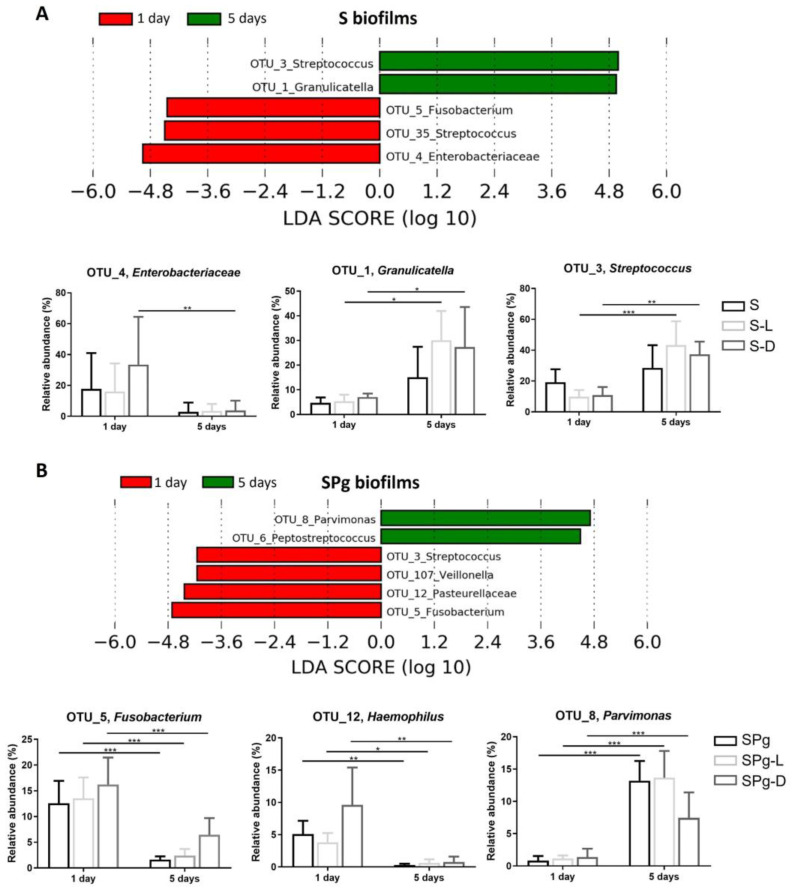
Linear discriminant analysis effect size (LEfSe) of OTUs that were differentially abundant in S biofilms (**A**) and SPg biofilms (**B**) as the biofilm aged. The default settings were used when performing LEfSe, except that the LDA threshold was set to 4. S biofilms, saliva-derived microcosms alone; SPg biofilms, saliva-derived microcosms spiked with *P. gingivalis*; L, intervention by peptide LL-31; D, intervention by peptide D-LL-31. Statistically significant differences are indicated by asterisks: * *p* < 0.05; ** *p* < 0.005; *** *p* < 0.0005 (two-way ANOVA followed by Bonferroni post hoc test).

**Figure 9:**
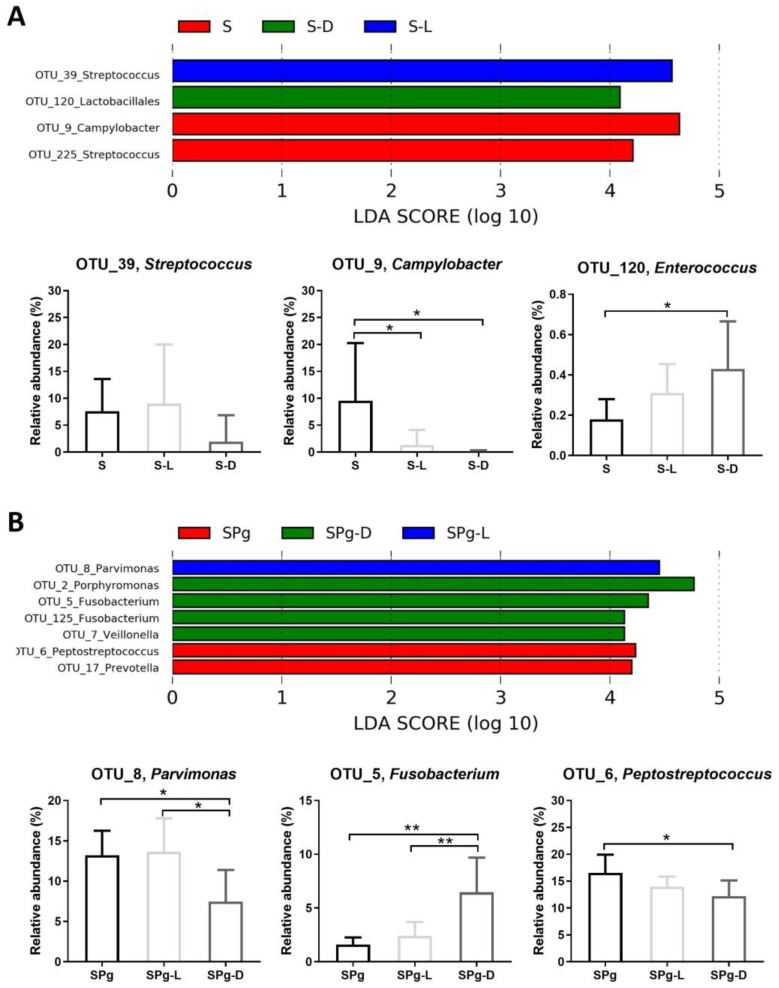
Linear discriminant analysis effect size (LEfSe) of OTUs that were differentially abundant between different treatment groups at 5 days after peptide treatments in S biofilms (**A**) and SPg biofilms (**B**). The default settings were used when performing LEfSe, except that the LDA threshold was set to 4 and one-against-all was used as the strategy for multi-class analysis. S biofilms, saliva-derived microcosms alone; SPg biofilms, saliva-derived microcosms spiked with *P. gingivalis*; L, intervention by peptide LL-31; D, intervention by peptide D-LL-31. Statistically significant differences are indicated by asterisks: * *p* < 0.05; ** *p* < 0.005 (one-way ANOVA followed by Bonferroni post hoc test).

LEfSe analysis further identified OTUs which were differentially abundant as biofilm aged (Figure 8), or among different treatment groups at 5 days after peptide intervention (Figure 9). In S biofilms (Figure 8A), the relative levels of abundance of OTU_1 (*Granulicatella*) and OTU_3 (*Streptococcus*) were significantly higher at day 5 than at day 1 after peptide treatments, whereas in SPg biofilms (Figure 8B), OTU_8 (*Parvimonas*) showed higher relative abundance in all treatment groups. Moreover, the relative abundance levels of OTU_5 (*Fusobacterium*) and OTU_12 (*Pasteurellaceae*) in SPg biofilms were significantly lower at day 5 than at day 1 in all treatment groups. Regarding the effect of peptide treatments on S biofilms at 5 days after intervention (Figure 9A), LL-31 and D-LL-31 treatment groups had a significantly decreased relative abundance of OTU_9 (*Campylobacter*) but an increased abundance of OTU_120 (*Lactobacillales*). As for SPg biofilms (Figure 9B), the D-LL-31 treatment group had a significantly lower relative abundance of OTU_8 (*Parvimonas*), but a higher relative abundance of OTU_5 (*Fusobacterium*).

## 4. Discussion

AMPs are small, diverse molecules that play critical roles in host defense and maintaining the host–microbe homeostasis [11]. Several AMPs, including defensins and cathelicidin, have been reported to function as microbiome modulators [12,14], working to restore a healthy gut microbiome. The current study investigated the microbiome modulation function of the potent variants of cathelicidin LL-37, namely, LL-31 and D-LL-31, using an in vitro microcosm biofilm model. Using the protocol established in a previous study [22], we successfully constructed two types of microcosm biofilms. The *P. gingivalis*-enriched microcosm biofilm contained 30% *P. gingivalis* and exhibited significantly higher protease activities than those of the microcosm biofilms with negligible level of *P. gingivalis*. The biofilm composition analysis also showed high abundance of proteolytic bacteria, including *Fusobacterium* and *Parvimonas*, indicating the more dysbiotic state of the SPg biofilms relative to the S biofilms. The 24 h D-LL-31 treatment significantly reduced the amount of *P. gingivalis*, total viable cell counts, and DPP4 activity of the SPg biofilms at 1 day after the treatment, but these effects diminished when the biofilm aged. LL-31 treatment did not show a noticeable effect on either of the biofilms. Different from the results of the biofilm functional test mentioned above, the altered microbial compositions of the biofilms caused by LL-31 and D-LL-31 treatments were still visible at 5 days after treatment.

In our initial bacterial growth inhibition test, LL-31 and D-LL-31 displayed clear antimicrobial activities against the periodontal pathogens *P. gingivalis* and *F. nucleatum*, but did not inhibit the growth of the commensal bacterium *S. mitis*. LL-31 seemed to be more potent than D-LL-31. Therefore, we hypothesized that both peptides might be able to modulate the *P. gingivalis*-enriched microcosm biofilms by inhibiting the growth of keystone pathogen *P. gingivalis*. However, differing from the results of growth inhibition test, we found that in microcosm biofilms, D-LL-31, not LL-31, was able to reduce the viability of *P. gingivalis* and the whole biofilm at 1 day after treatment. D-LL-31 is the D-enantiomeric form of LL-31 [21]. In natural AMPs, L-amino acids are present as the main amino acids but are prone to degradation by proteases in vivo [34], which potentially limits the clinical application of natural AMPs. Incorporation of D-amino acids in AMPs has been proposed as a solution to resist protease degradation and to improve the stability of AMPs without affecting their antimicrobial activity [35,36]. Hence, the different antimicrobial activities of LL-31 and D-LL-31 observed in single-species planktonic culture and microcosm biofilms might be related to the different susceptibilities of these two peptides to proteases. Likely, there were more proteases or higher protease activities in multi-species biofilms than found in single-species planktonic cultures. Consequently, D-LL-31 could function better than LL-31 in a multi-species biofilm environment. In a previous study, the gingipain of *P. gingivalis* displayed considerably higher activity when *P. gingivalis* grew in biofilms than when it grew as a planktonic culture [37].

The modulation of AMPs on the gut microbiome has been demonstrated by several studies [15,38], but this has been less studied in the oral microbiome. One pioneer study by Radaic, et al. [39] showed that nisin (a polycyclic AMP) and a nisin-producing probiotic *Lactococcus lactic* were able to modulate pathogenic oral biofilms towards health using an in vitro saliva-derived biofilm model. Similar to the current study, the saliva-derived biofilms in the study of Radaic, et al. [39] were also spiked with periodontal pathogens including *P. gingivalis*, and the duration of AMP treatment was 24 h as well. Both the study of Radaic, et al. [39] and this study showed that AMP could reduce either total biomass or total viable cell counts. However, Radaic, et al. [39] did not examine the long-term effect of nisin. The biofilm samples were collected right after treatments. Our study investigated the effects of LL-31 and D-LL-31 at 1 day and 5 days after treatments. It seems that the function of the peptides was affected by the dysbiotic state of the biofilms. In S biofilms, significant shifts of the microbial compositions could be observed at 5 days after the treatments of both peptides, whereas in SPg biofilms, which were the more dysbiotic biofilms, only D-LL-31 could cause a shift in microbial composition and the effect lasted only one day. Therefore, the modulating effects of LL-31 and D-LL-31 clearly depended on the dysbiotic state of the biofilms. The peptide function was short-term and was much less potent when treating the biofilms mimicking severe periodontitis.

As a keystone pathogen, *P. gingivalis* has a community-wide influence which is disproportionate to its abundance [40]. It was reported that oral inoculation of specific-pathogen-free (SPF) mice with *P. gingivalis* led to significant alteration in both the quantitative and qualitative composition of the commensal microbial community [41,42]. The data of the current study also demonstrated this orchestrating role of *P. gingivalis* in a microbial community: the spike of *P. gingivalis* altered the structure of the microcosm biofilms completely, which could be determined by the higher total viable cell counts, higher total protease and DPP4 activities, increased species richness and enriched obligate anaerobes such as *Parvimonas*. *P. micra*, often found in periodontitis patients, was originally classified as *Peptostreptococcus micros*, then reclassified as *Micromonas micros*, and reclassified again as *P. micra*. Previously, it has been shown that *P. micra* and *P. gingivalis* could co-aggregate and were found in close proximity to each other in a biofilm [43,44]. Nevertheless, the strong shift in microbial composition caused by *P. gingivalis* might explain why the modulating effect of D-LL-31 could not be observed, since it could only reduce 50% of *P. gingivalis* in the biofilms at 1 day after treatment (see Figure 3A).

In conclusion, our data illustrated that D-LL-31 had a better modulation effect as compared to LL-31 against saliva-derived microcosm biofilms enriched with *P. gingivalis*. Moreover, we also showed that the dysbiotic state of the biofilms could affect the modulation function of the peptides, since the duration and efficacy of the peptide function was clearly impaired in the *P. gingivalis*-enriched microcosm biofilms which mimic the biofilms in severe periodontitis.

## Figures and Tables

**Table 1:** One-way PERMANOVA statistical analysis of the influences of peptide treatments on the microbial compositions of biofilms.

Biofilm	Age	Treatment			*F* Value ^1^	*p* Value ^1^
**S**	1 day	no	vs.	LL-31	1.0	1.000
		no	vs.	D-LL-31	2.2	0.074
		LL-31	vs.	D-LL-31	0.6	1.000
	5 days	no	vs.	LL-31	**3.5**	0.008
		no	vs.	D-LL-31	**5.4**	0.002
		LL-31	vs.	D-LL-31	0.8	0.575
SPg	1 day	no	vs.	LL-31	2.0	0.071
		no	vs.	D-LL-31	**15.0**	0.0003
		LL-31	vs.	D-LL-31	**12.3**	0.0003
	5 days	no	vs.	LL-31	1.9	0.072
		no	vs.	D-LL-31	**8.8**	0.001
		LL-31	vs.	D-LL-31	**5.6**	0.001

^1^ *F* values and Bonferroni-corrected *p* values for the pairwise comparison in one-way PERMANOVA (Bray–Curtis distance; 9999 permutation). The effects of peptide treatments on each type of biofilms (S or SPg) at each time point (1 day or 5 days) were analyzed separately.

## Data Availability

The raw sequence and metadata generated has been submitted to the NCBI BioProject database under accession number PRJNA1031418.
